# Defects in the Ferroxidase That Participates in the Reductive Iron Assimilation System Results in Hypervirulence in *Botrytis Cinerea*

**DOI:** 10.1128/mBio.01379-20

**Published:** 2020-08-04

**Authors:** Esteban Vasquez-Montaño, Gustavo Hoppe, Andrea Vega, Consuelo Olivares-Yañez, Paulo Canessa

**Affiliations:** a Centro de Biotecnologia Vegetal, Facultad de Ciencias de la Vida, Universidad Andres Bello, Santiago, Chile; b Millennium Institute for Integrative Biology (iBio), Santiago, Chile; Cornell University

**Keywords:** *Botrytis cinerea*, ferroxidase, hypervirulence, iron uptake, multicopper oxidases, reductive iron assimilation

## Abstract

The plant pathogen Botrytis cinerea is responsible for gray-mold disease, which infects a wide variety of species. The outcome of this host-pathogen interaction, a result of the interplay between plant defense and fungal virulence pathways, can be modulated by various environmental factors. Among these, iron availability and acquisition play a crucial role in diverse biological functions. How *B. cinerea* obtains iron, an essential micronutrient, during infection is unknown. We set out to determine the role of the reductive iron assimilation (RIA) system during *B. cinerea* infection. This system comprises the BcFET1 ferroxidase, which belongs to the multicopper oxidase (MCO) family of proteins, and the BcFTR1 membrane-bound iron permease. Gene knockout and complementation studies revealed that, compared to the wild type, the *bcfet1* mutant displays delayed conidiation, iron-dependent sclerotium production, and significantly reduced whole-cell iron content. Remarkably, this mutant exhibited a hypervirulence phenotype, whereas the *bcftr1* mutant presents normal virulence and unaffected whole-cell iron levels and developmental programs. Interestingly, while in iron-starved plants wild-type *B. cinerea* produced slightly reduced necrotic lesions, the hypervirulence phenotype of the *bcfet1* mutant is no longer observed in iron-deprived plants. This suggests that *B. cinerea bcfet1* knockout mutants require plant-derived iron to achieve larger necrotic lesions, whereas *in planta* analyses of reactive oxygen species (ROS) revealed increased ROS levels only for infections caused by the *bcfet1* mutant. These results suggest that increased ROS production, under an iron sufficiency environment, at least partly underlie the observed infection phenotype in this mutant.

## Abstract (precis)

The plant-pathogenic fungus *B. cinerea* causes enormous economic losses, estimated at anywhere between $10 billion and $100 billion worldwide, under both pre- and postharvest conditions. Here, we present the characterization of a loss-of-function mutant in a component involved in iron acquisition that displays hypervirulence. While in different microbial systems iron uptake mechanisms appear to be critical to achieve full pathogenic potential, we found that the absence of the ferroxidase that is part of the reductive iron assimilation system leads to hypervirulence in this fungus. This is an unusual and rather underrepresented phenotype, which can be modulated by iron levels in the plant and provides an unexpected link between iron acquisition, reactive oxygen species (ROS) production, and pathogenesis in the *Botrytis*-plant interaction.

## INTRODUCTION

The ascomycete Botrytis cinerea belongs to the *Sclerotiniaceae* family, which comprises several fungal necrotrophs. Pathogenicity across a broad host range characterizes this mode of infection. Accordingly, the current survey of plants that can be infected by *B. cinerea* surpasses 1,000 hosts, and at least half of these are economically important (1, 2). As such, *B. cinerea* is an agrorelevant fungus, considered to be the second most crucial fungal phytopathogen worldwide (3). *B. cinerea* has caused enormous economic losses and has also served as a fundamental biological model for investigating the necrotrophic mechanisms of plant infection (4). Although intensively studied, there are still significant challenges to understand *B. cinerea* infection strategies (5). From a historical perspective, *B. cinerea* is considered a fearless and somewhat “brutal” plant killer (6, 7). Nevertheless, current evidence indicates the opposite, since subtle interactions between the pathogen and the host can occur (8), including the demonstration that *B. cinerea* produces cross-kingdom small RNA (sRNA) that targets plant immunity genes during infection (9–11). These and other newly revealed properties, such as the effect of light (reviewed in reference 12) and the circadian regulation of fungal virulence (13–15), exemplify novel aspects of *B. cinerea* pathogenesis (2).

The sessile nature of plants restrains the environment in which a plant-pathogen interaction develops. Thus, both the plant and the pathogen are confined to a specific location during the infection, and their biology is actively regulated by a series of environmental signals, including, but not limited to, light, temperature, pH, humidity, and nutrients, among others (16). Not surprisingly, nutrients are strong environmental cues. They play a key role in plant development and also in defense (17, 18). Among the nutrients that can most significantly constrain plant defense (19), there is a micronutrient that can set the difference in the arms race between a pathogen and its host: that environmental cue is iron.

Iron homeostasis is tightly linked to immunity and defense mechanisms in plants and throughout the tree of life (20, 21). The metal is fundamental for virulence in many microbial pathogens, including fungi (22, 23). Iron’s exceptional redox properties easily allow its transition between Fe(II) and Fe(III) (and *vice versa*), permitting critical biological functions within the cell that require electron transfer reactions, such as respiration and photosynthesis, among (several) others. Nevertheless, iron has conflicting characteristics. For instance, Fe(III) is insoluble in water, while Fe(II) is extremely soluble and prone to generate highly toxic reactive oxygen species (ROS) through the Fenton reaction (24). Although it is the second most abundant metal on the Earth’s crust, its bioavailability is limited under most aerobic conditions, including alkaline soils (25). Hence, organisms have evolved sophisticated methods to ensure homeostatic levels of the metal to fulfill critical functions. These systems are, therefore, interesting targets than can tilt the balance in the outcome of a plant-pathogen interaction.

Fungi can acquire iron using two strategies that can coexist in the same organism. The first approach consists of a transporter-mediated acquisition system of low-molecular-weight Fe(III)-siderophores and/or heme groups (26, 27), while the second strategy, known as reductive iron assimilation (RIA), relies on the acquisition of the metal through a plasma membrane system (28). *B. cinerea* is expected to produce at least nine siderophores, with ferrirhodin being the most abundant. *B. cinerea* can also take up five other known siderophores (29). Synthesized by complex nonribosomal peptide synthetases (NRPSs), the genome of *B. cinerea* encodes 11 NRPSs that may account for their synthesis. None of these have been experimentally validated but are predicted from *in silico* studies (30). In the biotrophic fungal plant pathogens Ustilago maydis and Microbotryum violaceum, the uptake of iron mediated by siderophores is dispensable for virulence (31, 32). In contrast, for the necrotrophic corn pathogen Cochliobolus heterostrophus, extracellular, but not intracellular, siderophores are required to display full virulence (33), highlighting the need of plant-derived iron to cause disease. On the other hand, the RIA system is considered the canonical fungal reductive pathway involved in iron assimilation (28). Initially described in Saccharomyces cerevisiae (34), RIA relies on the membrane-bound ferroxidase known as FET3, a member of the widely distributed family of multicopper oxidase (MCO) proteins (35, 36) that include four enzyme superfamilies: ascorbate oxidases, ceruloplasmins, laccases (phenol oxidases), and ferroxidases. FET3 facilitates metal uptake through its functional partner, FTR1 iron permease. In *U. maydis*, iron uptake via the combined action of the FET3 ferroxidase and the FTR1 permease that form the RIA system in this fungus (37) is required for virulence. This observation sharply differs from what has been described for *C. heterostrophus*, in which mutants lacking any of the RIA components do not display virulence alterations (38). As in the case of the NRPS-dependent iron uptake system that has not been studied in detail in *B. cinerea*, RIA has not been examined either. This highlights that in this particular phytopathogen, the impact of iron acquisition on virulence is unknown (39).

Although it has been suggested that iron content should be considered a strong environmental cue that modifies plant-pathogen interactions from the host-defensive perspective (20), iron uptake and traffic may also be important for the pathogen. Because very little is known about how *B. cinerea* obtains iron during infection, and considering that any significant perturbation in iron homeostasis will impact both plant immunity and pathogen virulence, we altered, by genetic disruption, the *B. cinerea* RIA system. Unexpectedly, although the ferroxidase (referred to here as BcFET1) mutant of the RIA system resulted in an iron-dependent phenotype, exhibiting significantly reduced whole-cell iron content during saprophytic growth, it was also found to exhibit augmented virulence. In contrast, the mutant for the iron permease (BcFTR1) did not reveal major iron-dependent phenotypes and displayed unaffected virulence. Our results suggest a role for BcFET1 in modulating Fe-dependent ROS generation and the effect of such regulation in impacting virulence.

## RESULTS

### Identification of an RIA-related ferroxidase-encoding gene (*bcfet1*) among multicopper oxidases of *B. cinerea*.

To explore the role of the reductive iron assimilation (RIA) system in *B. cinerea* infection dynamics, we searched for genes that encode orthologues of the FET3/FTR1 system in its genome database. As mentioned, both proteins constitute the canonical fungal iron uptake complex (34). Employing the FET3 protein sequence from S. cerevisiae as bait, BLAST analysis retrieved 13 protein sequences containing multicopper oxidase (MCO) domains. The first hit identified was *bclcc13* (described below). Therefore, we further analyzed the identified MCO sequences to distinguish ferroxidases among all identified proteins.

As indicated in Table S1 in the supplemental material, the identified MCO sequences possess annotated names that range from BcLcc1 to BcLcc13. A single MCO sequence was additionally identified when BcLcc1 to BcLcc13 were used as a BLAST analysis query (Bcin08g03600). The initials “Lcc” commonly refer to laccase enzymes, which are phenol oxidases that belong to the large family of MCO proteins described in different organisms, including plants and fungi (35, 36). Nevertheless, although similar, they are distinct from ferroxidases. In the case of *B. cinerea*, only two laccases have been studied in detail, BcLcc1 and BcLcc2, which were first described in this fungus several years ago (40). Hence, all the mentioned proteins were inspected using multiple-sequence alignments. First, we analyzed previously defined laccase signatures (41) that differ in MCOs with ferroxidase activity, such as FET3. As shown in Fig. S1, the L3 signature is conserved across all analyzed sequences. In contrast, the L1 signature differs in the cases of BcLcc6, BcLcc7, BcLcc8, BcLcc11, and BcLcc13, while L2 and L4 signatures were more conserved among BcLcc sequences ranging from BcLcc1 to BcLcc12. Notably, for BcLcc13, all signatures were more similar to those of the S. cerevisiae FET3 protein, making this protein a suitable RIA-related ferroxidase (and not a laccase) candidate for further inspection.

10.1128/mBio.01379-20.1 FIG S1 Laccase signatures in the MCO proteins identified in the *B. cinerea* genome. Laccase signatures from L1 to L4 (see the text) were analyzed for all identified MCO sequences in the *B. cinerea* genome. Residues that differ from the consensus sequence are denoted in red letters. L signatures are indicated at the top section of each box. BcLcc13 was termed here as BcFET1. For comparative purposes, the FET3 protein from S. cerevisiae was included. Download FIG S1, TIF file, 2.4 MB. Copyright © 2020 Vasquez-Montaño et al. 2020 Vasquez-Montaño et al. This content is distributed under the terms of the Creative Commons Attribution 4.0 International license.

10.1128/mBio.01379-20.10 TABLE S1 Multicopper oxidase (MCO) encoding genes of *B. cinerea*. The table shows 13 MCO sequences, including *bcfet1* as well as an MCO-like sequence not closely related to ferroxidases (ID Bcin08g03600). Download Table S1, DOCX file, 0.01 MB. Copyright © 2020 Vasquez-Montaño et al. 2020 Vasquez-Montaño et al. This content is distributed under the terms of the Creative Commons Attribution 4.0 International license.

**FIG 1:**
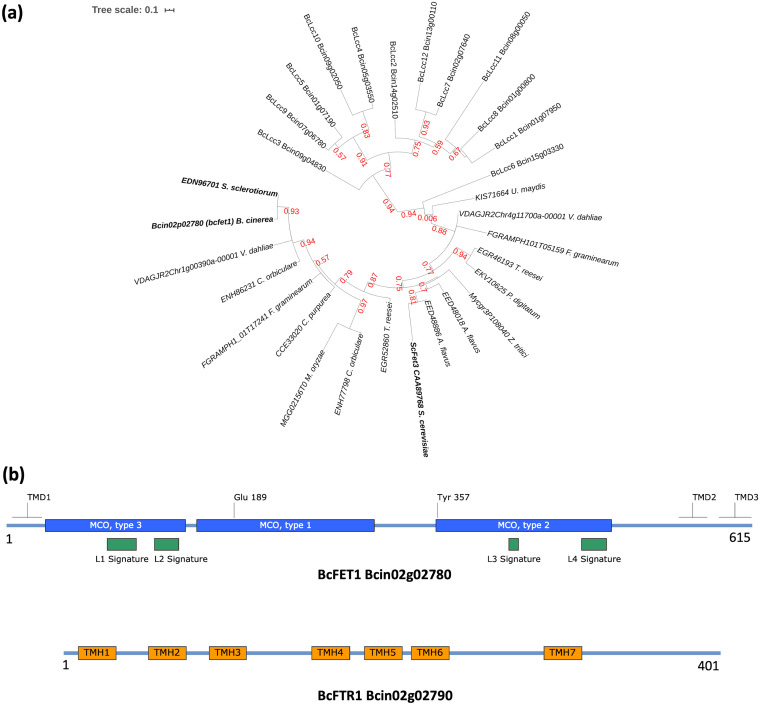
Computational analysis of the components of the *B. cinerea* RIA system. (a) Phylogenetic reconstruction of MCO proteins obtained from the *B. cinerea* genome. Amino acid sequences were acquired from different filamentous fungal pathogens, as mentioned in the text. The analysis was performed using the maximum likelihood method on the Phylogeny.fr platform. The tree denotes MCO sequences as BcLcc1-12 and Bcin02g02780 (BcFET1), followed by their corresponding unique IDs. S. cerevisiae and *S. sclerotiorum* FET3 sequences (ScFET3, EDN96701) are also indicated (in boldface; GenBank protein accession number CAA89768). Accession numbers were also retrieved from protein FASTA files available at Ensembl Fungi. (b) Schematic representation of BcFET1 (top) and BcFTR1 (bottom). The L1 to L4 signatures of BcFET1 (615 aa; see the text) are denoted in green boxes, while MCO domains 1, 2, and 3 (PFAM PF00394, PF07731, and PF07732, respectively) are indicated in blue boxes. Predicted transmembrane domains (TMD) are also indicated. In the case of BcFTR1 (401 aa), seven transmembrane helices (TMH) were predicted and are indicated in orange boxes. Each protein is depicted to scale.

Accordingly, a phylogenetic analysis employing the one-click method of the Phylogeny.fr platform was conducted (42). As observed in Fig. 1a, the phylogenetic reconstruction shows two major clades. In the first clade, all laccase MCO enzymes (BcLccs) of *B. cinerea* were clustered. In contrast, in the second, only BcLcc13/Bcin02g02780 (here termed BcFET1) grouped with the analyzed RIA-related ferroxidases, including those from different filamentous fungal plant pathogens. Compared to the FET3 protein from S. cerevisiae and Sclerotinia sclerotiorum, a closely related necrotrophic plant-pathogenic fungus, BcFET1 shares 47.5 and 87.2% amino acid sequence identity, respectively. According to the Conserved Domain Database (CDD) search (43), BcFET1 presents an MCO domain (COG2132) with three cupredoxin domains between amino acid residues 22 and 500 (Fig. 1b). Manual inspection of the alignment also allowed for the identification (in BcFET1) of a glutamic acid and a tyrosine residue equivalent to that of the FET3 protein from S. cerevisiae (185 and 354, equivalent to 189 and 357 in BcFET1; Fig. 1b). These two residues are critical for the ferroxidase activity of the protein (44). Collectively, and considering that the latter residues are absent from laccases, including BcLcc1 to BcLcc2, we conclude that *bclcc13* (referred to here as *bcfet1*) encodes an RIA-related ferroxidase, most likely involved in iron acquisition.

### Characterization of the RIA system of *B. cinerea*.

The open reading frame (ORF) of *bcfet1* comprises 1,848 bp, is interrupted by four introns, and encodes a 615-amino acid (aa) protein. Like its counterparts in many filamentous and nonfilamentous fungi (39), *bcfet1* is located in a genomic region close to its functional partner, here named *bcftr1*, which encodes an iron permease protein (gene identifier [ID] Bcin02g02790). As shown in Fig. S2, both *bcfet1* and *bcftr1* are 1,977 bp apart (between each translation start codon) and are divergent in transcriptional orientation. Computational analysis indicates the predicted BcFTR1 protein possesses 401 aa, shares 44% amino acid sequence identity with the FTR1 protein from S. cerevisiae, and presents the expected seven transmembrane helices (Fig. 1b, TMH; between aa residues 10 and 32, 53 and 75, 90 and 112, 153 and 175, 185 and 207, 214 and 236, and 295 and 317), as predicted by the TMHMM software (45). This allows the protein to channel ferroxidase (FET)-derived iron from the extracellular medium to the intracellular compartment. No paralog sequences were identified in the genome database.

10.1128/mBio.01379-20.2 FIG S2 Schematic representation of the *bcfet1*-*bcftr1 locus* in *B. cinerea*. Both are single-copy genes (IDs Bcin02g02780 and Bcin02g02790, respectively) located in chromosome 2. Arrows indicate their respective transcriptional orientation, sharing a divergent promoter region. Exons are denoted in green, while introns are in blue boxes. Download FIG S2, TIF file, 0.6 MB. Copyright © 2020 Vasquez-Montaño et al. 2020 Vasquez-Montaño et al. This content is distributed under the terms of the Creative Commons Attribution 4.0 International license.

Altogether, the provided *in silico* evidence strongly suggest the presence of an RIA system in *B. cinerea* that is encoded by gene IDs Bcin02g02780 and Bcin02g02790, composed of a single MCO gene encoding a BcFET1 ferroxidase and a functionally related gene encoding an iron permease, *bcftr1*.

### Generation of *bcfet1* deletion and complementation mutants.

To assess the influence of BcFET1 on *B. cinerea* iron responses and virulence, mutants with disrupted *bcfet1* activity were functionally characterized. For this purpose, we generated the Δ*bcfet1* mutant strain employing a homologous recombination strategy, which is illustrated in Fig. S3. Three independent Δ*bcfet1* deletion mutants with single integrations of the *hph* replacement cassette, as determined by quantitative PCR (qPCR) (see Table 2), were used. While all independent Δ*bcfet1* mutants exhibit equivalent phenotypes, an arbitrarily chosen mutant was subjected to genetic complementation to confirm the phenotypes associated with the deletion of *bcfet1*. Under the control of the endogenous wild-type *bcfet1*-*bcftr1* divergent promoter region (Fig. S2), *bcfet1* was targeted to the wild-type locus by homologous recombination. This yielded a hygromycin-sensitive and nourseothricin-resistant homokaryotic complementation mutant strain, termed Δ*bcfet1*+*bcfet1*. As in the case of the Δ*bcfet1* mutant strain, the homologous recombination of the complemented mutant was analyzed by PCR (Fig. S4).

10.1128/mBio.01379-20.3 FIG S3 Generation and genotypification of the Δ*bcfet1* strain. (a, bottom) Knockout strategy showing the *hph* replacement cassette (promoter region in green, coding sequence in blue) and the expected *in-locus* insertion of the genetic construct. (Top) The *bcfet1* gene and its transcriptional orientation (gene model ID Bcin02g02780) are represented as an orange arrow (small boxes denote introns). The position of the genomic regions employed for the homologous recombination (in red) and knockout generation are shown (to scale) next to *bcfet1*. Small black arrows show primers used for diagnostic PCRs and replacement cassette assembly, indicating their respective position and orientation. (b) Diagnostic PCRs. Homologous integration of a representative knockout strain at the 3′ (lane 1) and 5′ region (lane 2). No wild-type allele was observed in Δ*bcfet1* mutants after single-spore isolation (see Materials and Methods) (lane 3, primers pc08 and pc09) compared with the B05.10 wild-type strain (lane 5). (c) Primer pairs and their corresponding sequences. Primer pairs were used in diagnostic PCRs shown in panel b, as well as in the replacement cassette assembly shown in panel a. Their respective position and orientation are depicted in panel a. Download FIG S3, TIF file, 1.7 MB. Copyright © 2020 Vasquez-Montaño et al. 2020 Vasquez-Montaño et al. This content is distributed under the terms of the Creative Commons Attribution 4.0 International license.

10.1128/mBio.01379-20.4 FIG S4 Genetic complementation of the Δ*bcfet1* strain with the *bcfet1* gene. (a, top) Knock-in complementation strategy showing the *nat* resistance cassette (promoter region in green, coding sequence in blue) and the expected in-locus insertion of the genetic construct in the mentioned Δ*bcfet1* mutant genetic background. (Bottom) The *bcfet1* gene (depicted in orange) was inserted in the wild-type locus under the transcriptional control of its endogenous promoter (5´ flank and upstream sequence). The position of the genomic regions employed for the homologous recombination (in red) and knock-in generation are shown (to scale) and correspond to the same sequences depicted in Fig. S3. Black arrows show primers used for diagnostic PCRs and complementation genetic cassette assembly, indicating their respective position and orientation. (b) Diagnostic PCRs. Homologous integration of a representative complemented mutant, termed Δ*bcfet1*+*bcfet1*, is indicated in lane 1. No *hph* PCR amplicon was detected for the Δ*bcfet1*+*bcfet1* complemented mutant strain (lane 2), while an 803-bp PCR amplicon (primer pair pc08 + pc09; indicated in Fig. S3), indicative of *bcfet3*, was detected in Δ*bcfet1*+*bcfet1* (lane 5) but not in Δ*bcfet1* (lane 4). (c) Primer pairs used in diagnostic PCRs shown in panel b, as well as in the complementation genetic construct assembly shown in panel a. Their respective position and orientation are depicted in panel a. Additional primers are indicated in Fig. S3. Download FIG S4, TIF file, 1.7 MB. Copyright © 2020 Vasquez-Montaño et al. 2020 Vasquez-Montaño et al. This content is distributed under the terms of the Creative Commons Attribution 4.0 International license.

### Deletion of *bcfet1* affects regular conidium formation.

**FIG 2:**
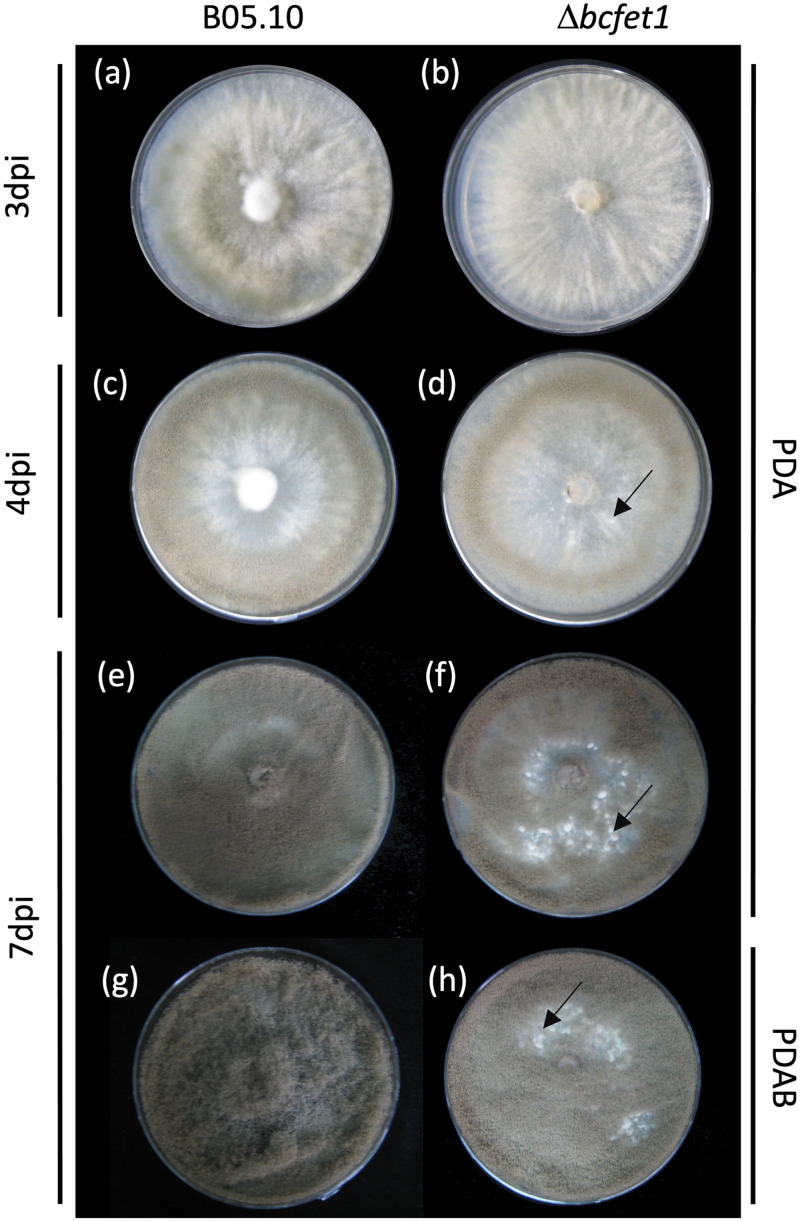
Deletion of *bcfet1* delays conidial production in *B. cinerea*. B05.10 and Δ*bcfet1* strains were inoculated as mycelial agar plugs on PDA or PDA-planta solid medium for the indicated periods (left). Primordia of sclerotia structures were observed only for the Δ*bcfet1* strain (black arrows in panels d, f, and h). Representative pictures are shown.

As observed in Fig. 2, the Δ*bcfet1* strain displays a reduced and delayed conidiation pattern compared to that of the B05.10 wild-type strain (Fig. 2a to d), which was determined as early as 3 and 4 days postinoculation (dpi) and also was supported by conidium quantification (Fig. S5). No significant differences in growth rate were observed after 3 days of growth, as depicted in Fig. S6. Interestingly, in addition to the reduced conidiation phenotype, at 4 dpi the Δ*bcfet1* strain started displaying primordia of sclerotia, which was observed as small white structures located near the inoculation site at the center of the petri dish (Fig. 2d). These structures were clearly observed 7 dpi (Fig. 2f and h). After 7 days of cultivation in regular PDA medium, the B05.10 wild-type strain presents the expected conidiation pattern (Fig. 2e) covering the full cultivation area, while in the presence of plant-supplemented PDA media (PDAB), the B05.10 strain displays the well-known profuse conidiation pattern (Fig. 2g). In contrast, although the Δ*bcfet1* strain can produce conidia under both culture conditions, primordia of sclerotium structures are still observed. Notably, these structures were detected when employing the regular photoperiod commonly used to grow *B. cinerea* (see Materials and Methods), a culture condition that favors conidium, but not sclerotium formation, the latter being favored in the absence of light (13).

10.1128/mBio.01379-20.5 FIG S5 Quantification of conidia production in *B. cinerea*. B05.10 and Δ*bcfet1* strains were inoculated on PDA. After 3 and 4 dpi, agar plugs were retrieved from the inoculated plates and resuspended in 1 ml sterile water. After filtration, conidia were quantified (*n* = 9) using a Thoma chamber. Mean values ± SE are shown in orange, as well as each independent measurement. Statistical differences (*P* < 0.05) are indicated with letters (different letters indicate significant differences). Download FIG S5, TIF file, 1.8 MB. Copyright © 2020 Vasquez-Montaño et al. 2020 Vasquez-Montaño et al. This content is distributed under the terms of the Creative Commons Attribution 4.0 International license.

10.1128/mBio.01379-20.6 FIG S6 Quantification of *B. cinerea* growth on solid media. B05.10, Δ*bcfet1*, and Δ*bcftr1*strains were inoculated on PDA as agar plugs. The area of growth was determined daily after 1, 2, and 3 dpi. Mean values ± SE are shown (*n* = 5). No statistical differences were determined. Download FIG S6, TIF file, 1.5 MB. Copyright © 2020 Vasquez-Montaño et al. 2020 Vasquez-Montaño et al. This content is distributed under the terms of the Creative Commons Attribution 4.0 International license.

### The deletion of *bcfet1* leads to sclerotium formation and reduces whole-cell iron content.

**FIG 3:**
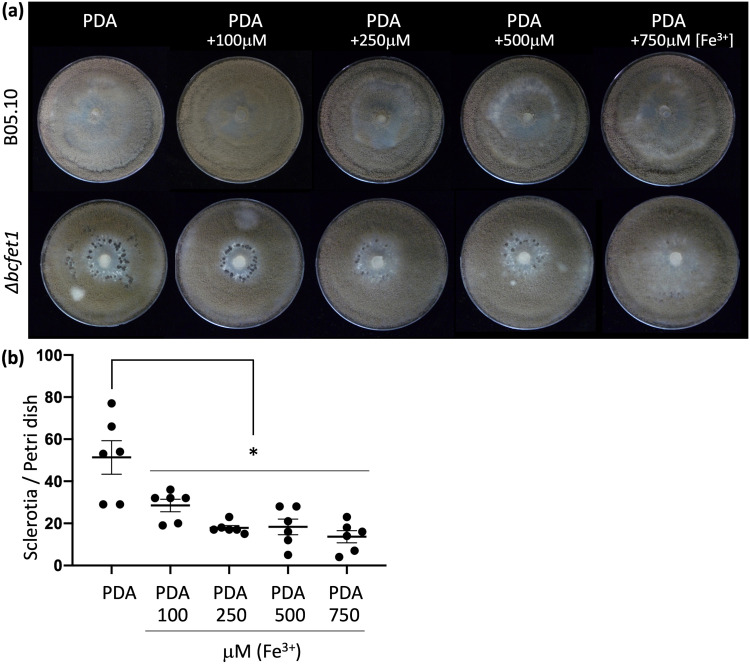
Deletion of *bcfet1* leads to sclerotium formation in an iron-dependent manner. (a) The B05.10 wild-type strain, as well as the Δ*bcfet1* strain, were inoculated on PDA or iron-supplemented (Fe^3+^) PDA medium for 10 days (final concentration is indicated). Cultures were incubated as indicated in Materials and Methods. Representative pictures are shown. (b) Quantification of sclerotia production by the Δ*bcfet1* strain (mean values ± standard errors [SE]). As expected, the wild-type strain did not produce sclerotia under the employed culture conditions. Statistical differences (*P* < 0.05) are indicated with an asterisk after pairwise comparisons.

To investigate whether the white structures depicted in Fig. 2 lead to the development of regular dark-pigmented sclerotia, longer cultivation times were analyzed. As shown in Fig. 3, sclerotia were observed after 10 dpi for the Δ*bcfet1* mutant but not the B05.10 wild-type strain. These structures are required for overwintering, enabling the fungi to survive unfavorable conditions during extended periods of time when temperatures and/or nutrient availability are low (46). This observation is consistent with a mutant strain bearing a reduced/impaired acquisition of iron. Therefore, we tested whether the supplementation of the culture media with this micronutrient reduced sclerotium formation. Importantly, in the tested culture conditions, the wild-type strain does not produce sclerotia (Fig. 3a, top). As shown in Fig. 3a, the Δ*bcfet1* strain showed a reduced number of sclerotia in the iron-supplemented PDA medium, and these significant differences were dependent on iron concentration (Fig. 3b). As definitive proof that the absence of *bcfet1* produces iron scarcity, we measured fungal iron content (see Materials and Methods). As shown in Fig. S7, after 3 dpi, the Δ*bcfet1* strain displayed a significantly reduced whole-cell iron content compared to that of the B05.10 wild-type strain. Finally, as shown in Fig. S8, the expression of *bcfet1* in the Δ*bcfet1* genetic background reverted the sclerotium phenotype seen in the Δ*bcfet1* mutant strain, although the complemented mutant failed to display normal levels of conidiation (see Discussion).

10.1128/mBio.01379-20.7 FIG S7 Whole-cell iron (Fe^3+^) content in the Δ*bcfet1* and Δ*bcftr1* strains. After 4 dpi, mycelia were recollected and subjected to iron (Fe^3+^) quantification (mean values ± SE; ppm/g) as indicated in Materials and Methods. Significant differences (*P* < 0.05) are indicated with asterisks. Download FIG S7, TIF file, 1.6 MB. Copyright © 2020 Vasquez-Montaño et al. 2020 Vasquez-Montaño et al. This content is distributed under the terms of the Creative Commons Attribution 4.0 International license.

10.1128/mBio.01379-20.8 FIG S8 Expression of *bcfet1* in the Δ*bcfet1* genetic background does not allow sclerotia formation. (a) B05.10, Δ*bcfet1*, and Δ*bcfet1*+*bcfet1* strains were inoculated on PDA medium, as indicated in Materials and Methods. Representative pictures are shown after 21 days of cultivation. (b) Magnification of the central region of the petri dish shown in panel a. Download FIG S8, TIF file, 2.5 MB. Copyright © 2020 Vasquez-Montaño et al. 2020 Vasquez-Montaño et al. This content is distributed under the terms of the Creative Commons Attribution 4.0 International license.

### The absence of *bcfet1* leads to a hypervirulence phenotype that depends on the plant’s iron status.

**FIG 4:**
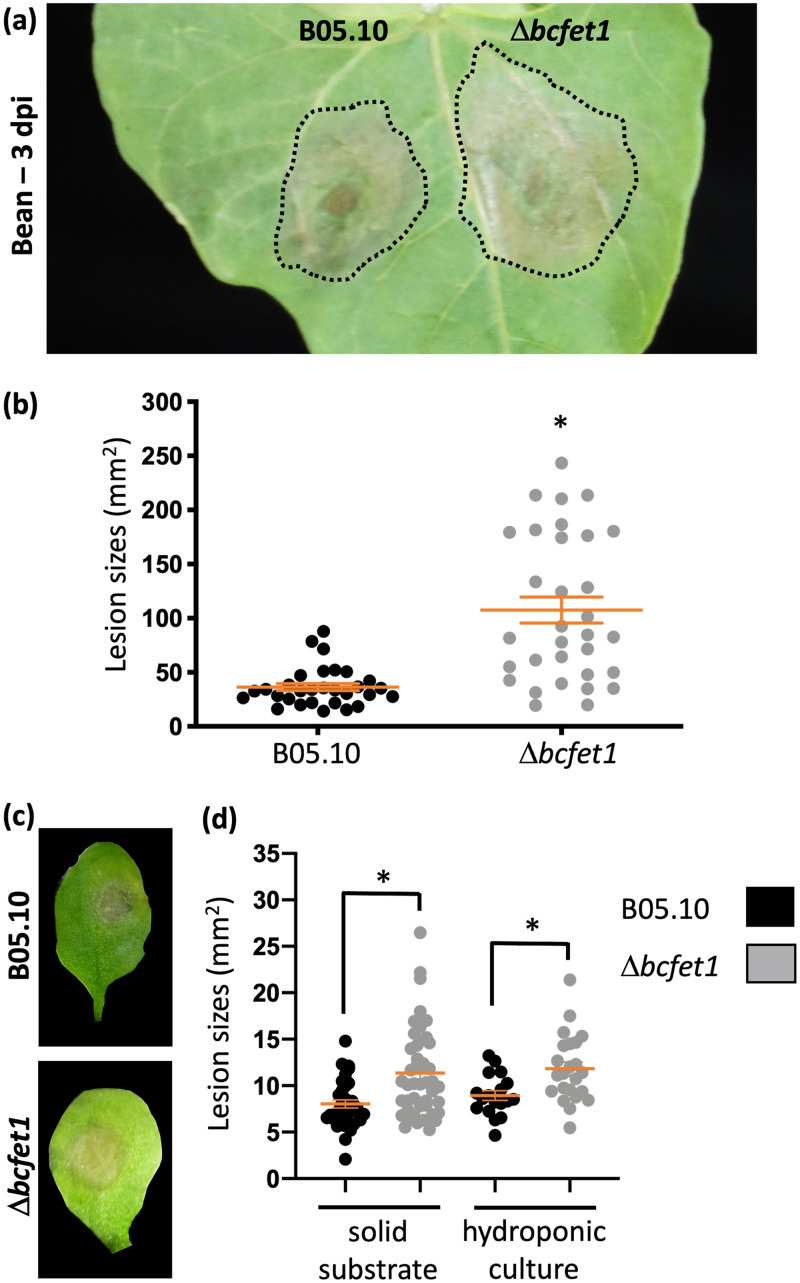
*B. cinerea* virulence is enhanced in the Δ*bcfet1* genetic background. (a) Lesion spreading of the Δ*bcfet1* mutant is significantly enhanced compared with that of the B05.10 wild-type strain. Primary leaves of living P. vulgaris plants were inoculated with conidial suspensions (7 μl of conidial suspensions of 2 × 10^5^ spores/ml) and incubated for 3 days in a humid chamber. (b) Lesion area (mean values ± SE; orange lines) as well as each independent measurement (scatter dot plot) were calculated employing three independent Δ*bcfet1* mutants. (c) Lesion spreading of Δ*bcfet1* mutants (inoculated as mentioned for panel a) in *A. thaliana* living plants grown in a solid substrate. (d) Lesion areas (mean values ± SE; orange lines) as well as each independent measurement (scatter dot plot) were calculated from *Arabidopsis* pathogenicity tests, employing both solid substrate and hydroponic cultures. At least four plants were used in each case. Statistical differences (*P* < 0.05) are indicated with asterisks.

To determine the relevance of BcFET1 and the RIA system during *B. cinerea* infection, we assayed virulence of the Δ*bcfet1* strain on French bean (Phaseolus vulgaris) and Arabidopsis thaliana Col-0 (Columbia 0) plants. These organisms represent highly and moderately *Botrytis*-susceptible hosts, respectively. As observed in Fig. 4, significantly increased necrotic lesions were found in both P. vulgaris and *A. thaliana* plants that were incubated for 3 days with the Δ*bcfet1* strain compared to the level for the B05.10 wild-type strain. In each case, plants were grown on a solid substrate mixture (details are in Materials and Methods). To properly control plant micronutrient availability, *A. thaliana* plants were grown hydroponically for 5 weeks and contained 50 μM FeNa-EDTA as the only iron source, a strategy that has been previously reported (47). Consistent with the results obtained using French beans and *A. thaliana* grown on a solid substrate, hydroponically grown *Arabidopsis* plants that were grown under sufficient iron concentrations also displayed a significantly increased necrotic lesion when inoculated with the Δ*bcfet1* strain (Fig. 4d).

**FIG 5:**
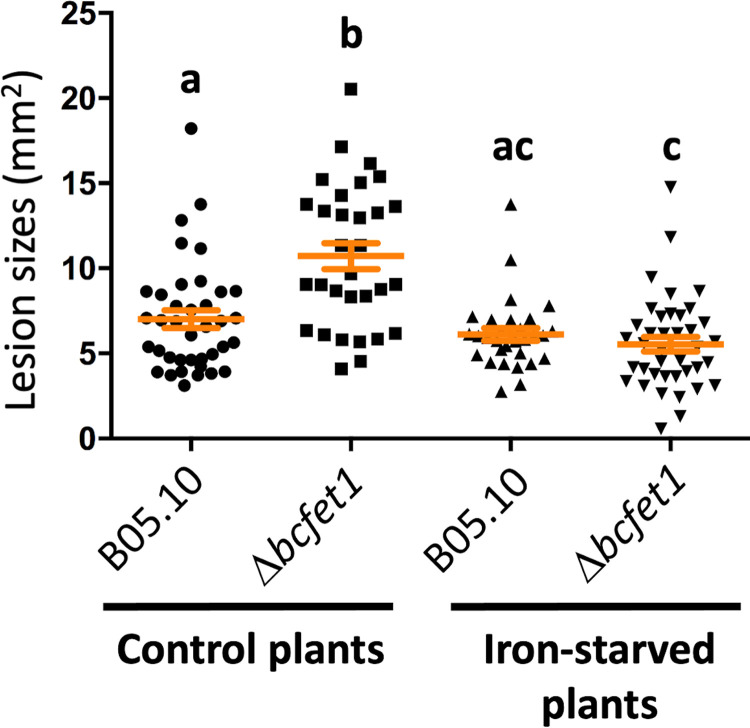
Δ*bcfet1* strain displays reduced virulence when infecting iron-deprived plants. The lesion spreading of the Δ*bcfet1* mutant is significantly reduced under iron-deprived (plant) conditions. *A. thaliana* living plants grown under sufficient iron conditions (control) or iron-starved conditions were inoculated with conidial suspensions (as indicated in Fig. 4) and incubated for 3 days (see Materials and Methods for details). The lesion area is depicted as mean values ± SE (in orange lines) as well as each independent measurement (scatter dot plot) calculated from at least 30 lesions per culture condition. Statistical differences (*P* < 0.05) are indicated with letters (different letters indicate significant differences).

Taking advantage of the aforementioned hydroponic culture conditions, *A. thaliana* plants were subjected to an iron deprivation protocol (47) that used bathophenanthroline disulfonic acid (BPDS; see Materials and Methods), a highly specific iron chelator. Importantly, this method allows for normal *Arabidopsis* development after 4 weeks of cultivation; at this time, iron-deprived plants were obtained and used for virulence assays. As shown in Fig. 5, the iron deprivation procedure did not significantly affect the observed lesion area caused by the B05.10 wild-type strain. In contrast, when employing iron-starved plants, a significant reduction in the necrotic lesion caused by the Δ*bcfet1* strain was clearly observed, indicating that the hypervirulence phenotype determined for this particular mutant strain depends on the plant’s iron availability.

### The *Δbcftr1* strain displays a wild-type level of infection.

According to the Pathogen-Host Interaction (PHI) database (48), the majority of the *B. cinerea* mutant strains that have been characterized display reduced or unaltered virulence. Increased lesions are seldom observed, which makes the Δ*bcfet1* phenotype all the more interesting. Thus, to better analyze this very rare infection phenotype, the RIA system of *B. cinerea* was further characterized. To this end, two independent *bcftr1* deletion mutants (Fig. S9) containing a single integration of the homologous *hph* recombination cassette (see Table 2) were obtained and characterized.

10.1128/mBio.01379-20.9 FIG S9 Generation and genotypification of the Δ*bcftr1* strain. (a, bottom) Knockout strategy showing the *hph* replacement cassette (promoter region in green, coding sequence in blue) and the expected in-locus insertion of the genetic construct. (Top) The *bcftr1* gene and its transcriptional orientation (gene model ID Bcin02g02790) are represented as an orange arrow (small boxes denote introns). The position of the genomic regions employed for the homologous recombination (in red) and knockout generation are shown (to scale) next to *bcftr1*. Small black arrows show primers used for diagnostic PCRs and replacement cassette assembly, indicating their respective position and orientation. (b) Diagnostic PCRs. Homologous integration of a representative knockout strain at 5’ (lane 1) and 3’ (lane 2) regions. No wild-type allele was observed in Δ*bcftr1* mutants after single-spore isolation (see Materials and Methods) (lane 4; primers pc55 and pc56) compared with the B05.10 wild-type strain (lane 5). (c) Primer pairs and their corresponding sequences are indicated. Primer pairs were used in diagnostic PCRs shown in panel b, as well as in the replacement cassette assembly shown in panel a. Their respective position and orientation are depicted in panel a. Download FIG S9, TIF file, 2.5 MB. Copyright © 2020 Vasquez-Montaño et al. 2020 Vasquez-Montaño et al. This content is distributed under the terms of the Creative Commons Attribution 4.0 International license.

**FIG 6:**
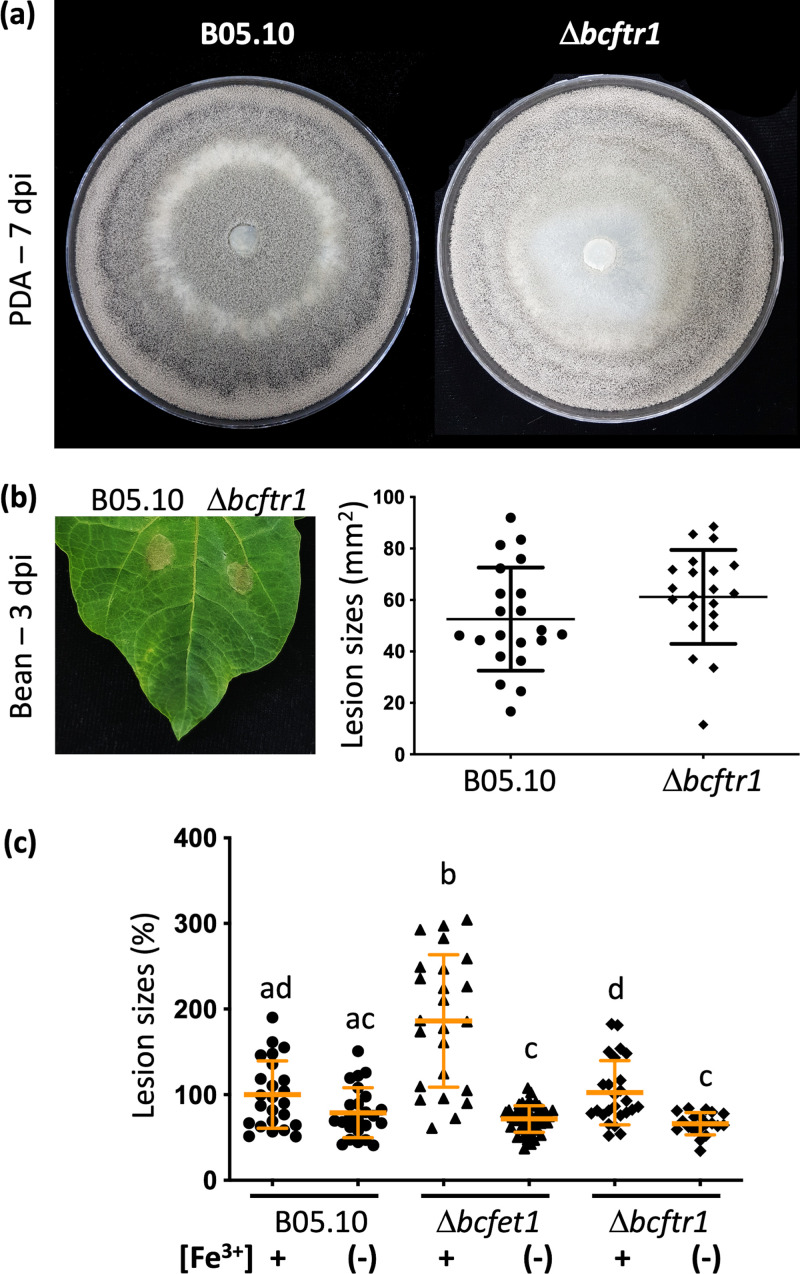
Δ*bcftr1* strain displays reduced virulence in iron-deprived plants but not hypervirulence in the presence of iron. (a) *In vitro* characterization of the *Δbcftr1* strain growing on PDA medium. (b) Lesion spreading areas (mean values ± SE) as well as each independent measurement (scatter dot plot) of the Δ*bcftr1* mutant on bean primary leaves. P. vulgaris leaves were inoculated with conidial suspensions (as indicated in Fig. 4) and incubated for 3 days, as indicated in Materials and Methods. (c) Lesion spreading areas on *A. thaliana* leaves, depicted as mean values ± SE (orange lines), as well as each independent measurement (scatter dot plot) calculated from at least 30 lesions per culture condition. *A. thaliana* living plants grown under sufficient iron conditions (Fe[+]) or iron-starved conditions (Fe[−]) were employed. For comparative purposes, values are referred to as the lesion caused by the wild-type strain in plants obtained under iron-sufficient conditions. Statistical differences (*P* < 0.05) are indicated with letters (different letters indicate significant differences).

Similar to the Δ*bcfet1* strain, the *bcftr1* mutant strain displays a reduced conidiation pattern that was clearly observed at the petri dish inoculation site (at the center of the plate). Nevertheless, no sclerotium formation was detected (Fig. 6a). Thus, this phenotype can be considered less severe than that observed from the *Δbcfet1* strain, although the *Δbcftr1* mutant also shows reduced whole-cell iron content (Fig. S7). In contrast to the Δ*bcfet1* strain, the Δ*bcftr1* mutant displayed a wild-type level of infection in P. vulgaris plants (Fig. 6b). Taking advantage of the *Arabidopsis* iron deprivation procedure mentioned above, we also analyzed the virulence of the Δ*bcftr1* mutant strain using hydroponically grown plants. As observed in Fig. 4, a 2-fold increase in the necrotic lesion area was observed for the Δ*bcfet1* mutant (Fig. 6c) only in *Arabidopsis* plants obtained under sufficient iron concentrations, whereas the Δ*bcftr1* strain failed to do so. In contrast, the Δ*bcftr1* strain was found to display a wild-type level of infection in the case of iron-supplemented plants and resulted in significantly reduced lesions when infecting iron-deprived plants (Fig. 6c).

**FIG 7:**
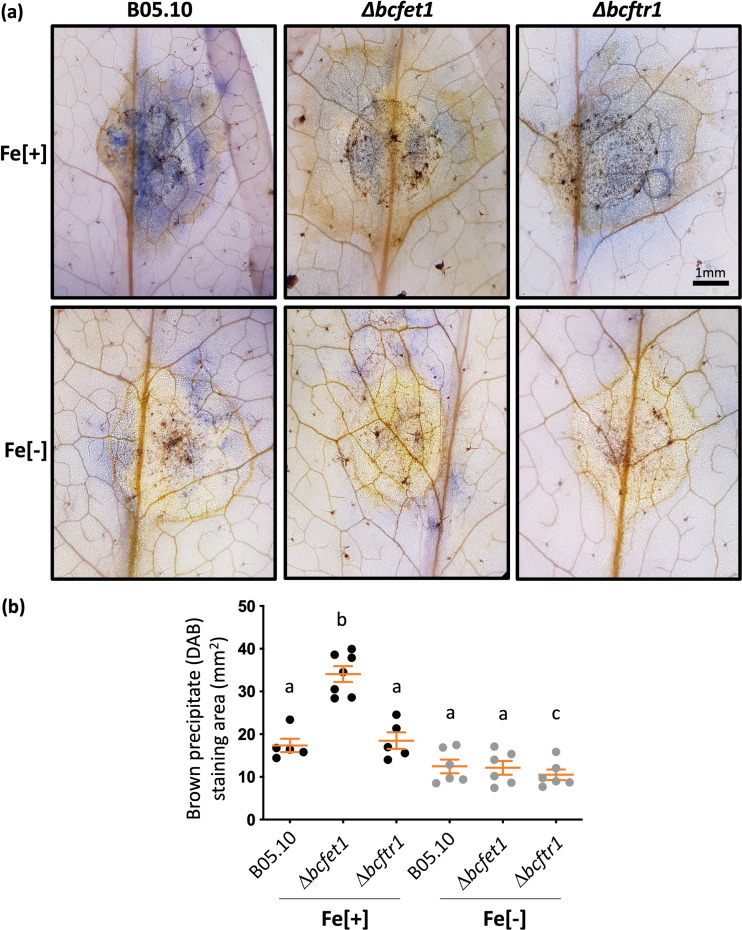
Leaves of *A. thaliana* plants infected with the *Δbcfet1* strain, but not the *Δbcftr1* strain, display a larger area of infection, indicative of ROS in the presence of iron. (a) Through DAB and cotton blue staining, the formation of a brown precipitate (indicative of ROS) and fungal progress was monitored, respectively. For comparative purposes, the lesion caused by the B05.10 wild-type strain was also analyzed. Fe[+], leaves obtained from *Arabidopsis* plants grown under iron sufficiency; Fe[−], leaves obtained from *Arabidopsis* plants grown under iron-limiting conditions. Infections were analyzed after 3 dpi. Representative pictures are shown. (b) Quantification of the area displaying DAB staining on *Arabidopsis* leaves infected with *B. cinerea*, as depicted in panel a. Each analyzed fungal strain was inoculated as indicated in the legend to Fig. 4. The area of the leaves exhibiting the formation of a brown precipitate (indicative of ROS) was quantified as indicated in Materials and Methods. Mean values ± SE are shown, as well as each independent measurement (*n* = 5). Statistical differences (*P* < 0.05) are indicated with letters (different letters indicate significant differences).

Considering that the infection phenotypes displayed by both RIA mutants of *B. cinerea* differ significantly, and because ROS is a hallmark of the plant defense responses, *B. cinerea* virulence (49), and iron homeostasis (50), we analyzed potential differences in the ROS levels caused by these two mutants during infection. To observe the oxidative burst (H_2_O_2_ production) generated on the plant tissue infected by *B. cinerea* (51), DAB staining was performed. In addition, fungal structures were stained using cotton blue, which allows for fungal chitin staining (52). As observed in Fig. 7, a diminished level of DAB staining was found for all *B. cinerea* strains when infection assays were carried out on iron-starved plants (Fig. 7a, bottom). In agreement with this observation, *in planta* hyphal growth (determined with cotton blue staining) was also strongly reduced in iron-deprived plants. In contrast, and consistent with the quantification of the necrotic lesion area determined above, both B05.10 and Δ*bcftr1* strains developed a similar brown-stained area when infecting *Arabidopsis* plants obtained under iron sufficiency conditions (Fig. 7b). On the other hand, the Δ*bcfet1* strain consistently developed a larger area (2-fold increment; Fig. 7b) of DAB staining, but only when infecting iron-supplemented *Arabidopsis* plants.

## DISCUSSION

Iron and the acquisition systems of this micronutrient have been described as modulators of infectious capacity. This includes different pathogens in general and phytopathogens in particular, including various fungi (36). However, iron capture systems in *B. cinerea* have not been studied in detail (39). The exceptions correspond to the results presented here and the identification of the major siderophores produced by this fungus, which were described several years ago, as well as their putative biosynthesis pathways, determined by computational predictions (29, 30). For these reasons, at least from a fungal perspective, little is known about how this metal modulates the virulence of this remarkable necrotrophic phytopathogen. While the relevance of iron on plant defense responses has been investigated (see below), the role of the metal on *B. cinerea* virulence is just beginning to emerge.

Iron significantly affects plant defense responses to bacterial and fungal necrotrophic phytopathogens, including *B. cinerea* (21, 47). The metal is an active player that participates in the defense response of *A. thaliana*, a plant that, when challenged with a pathogen, modifies iron uptake and mobilization (53–55). In this regard, at least two plant phytohormones, salicylic acid (SA) and jasmonic acid (JA), constitute major signaling pathways involved in plant defense and have a substantial impact on the plant’s iron homeostasis. Generally speaking, SA is involved in the signaling of defense mechanisms against biotrophic pathogens (56), while JA is considered the main phytohormone responsible for inducing the defense response to necrotrophs (57, 58). The latter phytohormone, among the orchestrated plant defense responses, decreases iron capture (at the rhizosphere level) in *Arabidopsis* by downregulating the expression of *FRO2* and *IRT1*, two genes that encode an iron uptake system. This strategy seeks to reduce the amount of iron that the pathogen can potentially acquire during infection (21, 54). Consistent with these investigations and others (47), we found a reduced necrotic lesion when the B05.10 wild-type strain infects iron-starved plants, an observation that was even more pronounced (and statistically significant) in the case of both RIA knockout mutants of *B. cinerea* (Fig. 6). These results indicate the metal and the RIA system are necessary (from a fungal perspective) during plant infection. In agreement with these observations, a reduction in the leaf area that displays the oxidative burst during plant colonization was also determined but only in iron-deprived *Arabidopsis*. When the metal is not limited, however, it is reasonable to expect that, from the Δ*bcfet1* strain that is deficient in iron acquisition (as demonstrated *in vitro*), there will be reduced necrotic lesions compared to the wild-type strain or even similar necrotic lesions, if the absence of *bcfet1* is compensated (e.g., siderophores), but not increased lesions, like those observed here.

Irrespective of the infection strategy employed by plant pathogens, both biotrophs and necrotrophs need iron to grow and survive during the infection. If the host withholds iron, virulence will decrease in both cases, although the plant needs iron to create an oxidative defense burst. In fact, SA increases iron uptake in *Arabidopsis* (21). However, necrotrophs like *B. cinerea* favor an oxidative environment, since it provides them with an advantage over biotrophs (49, 59). In this regard, the mechanisms of iron acquisition employed by different pathogenic fungi seem to be closely related to their infection strategy, as previously noted (38). The uptake of iron mediated by siderophores has been described as fundamental for virulence in necrotrophic phytopathogens such as Alternaria brassicicola, Alternaria alternata, *C. heterostrophus*, F. graminearum, and other opportunistic mammalian pathogens that destroy the host cell, such as Aspergillus fumigatus (33, 38, 60–62), and even entomopathogenic fungi (63). In contrast, biotrophic organisms that, unlike necrotrophs, require a living host cell to infect have been found to need the RIA system for iron acquisition during plant infection (see below), an observation that differs from the results reported here for *B. cinerea*.

Importantly, the complemented Δ*bcfet1*+*bcfet1* mutant clearly indicates that the expression of *bcfet1* is required to inhibit sclerotium formation, structures that are no longer developed in the Δ*bcfet1* genetic background when the metal is added to the culture media. Nevertheless, the complemented mutant failed to restore regular conidiation, indicating that proper *bcfet1* expression also is required for fungal morphogenesis and development. Considering that iron acquisition in fungal biological systems is delicately and tightly controlled at multiple levels, including the regulation of transcription (28, 39), the strategy utilized to complement the wild-type copy back in the mutant background may have altered this. Indeed, based on the divergent transcriptional orientation of the two genes under analysis, it was not possible to employ the *nat* resistance cassette upstream of the 5′ recombinational flank of *bcfet1*, in which case the divergent promoter region would have been interrupted. However, localizing the *nat* resistance cassette in the 3′ region of the introduced copy of *bcfet1* may have altered proper termination or mRNA accumulation. Nevertheless, the complementation strategy did allow us to confirm the observed hypervirulence effect associated with the absence of *bcfet1* in the corresponding mutant.

The two most completely analyzed MCOs from *B. cinerea* are the laccases BcLcc1 and BcLcc2 (40). These types of enzymes can detoxify plant-derived antifungal and antimicrobial compounds such as phytoalexins (64), although *bclcc2* loss-of-function mutants display wild-type levels of virulence (40), which sharply contrasts with the Δ*bcfet1* strain. The infection phenotype of this particular mutant strain also differs from those of other fungal pathogens in which the RIA system has been studied. Components of the RIA in different fungal pathogens have been described as crucial for observing their full pathogenic potential, which contrasts with the hypervirulence phenotype described here for the Δ*bcfet1* strain. This is the case for Candida albicans (65), in which RIA mutants display reduced virulence. In the case of the maize-specific biotrophic plant pathogen *U. maydis*, in the absence of the RIA system, it exhibited only a reduction in symptoms and plant lesions (37). However, the fungus still manages to cause disease, indicating that reductive iron assimilation is required to display full virulence. The same observation is valid for the biotrophic plant smut fungi *M. violaceum* (32). More recent investigations performed in the hemibiotrophic fungus *Colletotrichum graminicola* support the idea that the ferroxidase/permease system is required for virulence in biotrophic infections. In this fungus, *fet3* is dispensable for the development of necrotic lesions on wounded plants, but the gene is required for the appressoria function, a key virulence determinant required for penetration of healthy plant tissue (66). This investigation and others (67) have provided evidence indicating that during the biotrophic phase of infection, RIA is used. When the fungus switches to the necrotrophic phase, siderophores are required for lesion development. Finally, recent investigations have provided additional perspectives for RIA. In *Paracoccidioides* species, whose genome does not encode an FTR1 iron permease, the authors suggested that the fungus utilizes a nonclassical FTR1-independent RIA system that requires Fe/Zn permeases, known as Zrts, that may account for iron uptake (68), adding an additional layer of complexity.

In S. cerevisiae, the FET3 and FTR1 proteins are not independent. They physically interact at the cytoplasmic level, forming a primary protein complex and subsequently comigrating to the plasma membrane, as demonstrated using fluorescence-tagged versions of each protein (69). In S. cerevisiae, it has been shown that the absence of the FTR1 protein causes a decrease in the migration of FET3 to the membrane with a fraction accumulating in cytoplasmic compartments. Similarly, in the absence of FET3, a partial migration of the iron permease to the membrane has been described (69). While this exemplifies the high level of functional interdependence necessary for metal uptake, it also provides genetic evidence that the components of the RIA system of *B. cinerea* are more autonomous than their yeast counterparts, since both RIA mutants display distinct (infection) phenotypes. As a multicopper ferroxidase that requires three Cu^2+^ atoms (70), it has been reported that the yeast FET3 apoprotein is not adequately assembled and is unable to migrate to the membrane (69). The copper atom donation is carried out by the copper transport ATPase termed CCC2 (71), whose ortholog gene in *B. cinerea* has been designated *bcccc2.* This gene is required for copper incorporation, but importantly, the Δ*bcccc2* mutant is not found to be deficient in iron incorporation (72), which strongly suggests that the fungus possesses functionally alternative mechanisms to acquire iron. However, the precise biochemical pathways involved have not been experimentally validated yet (29, 30).

According to the Pathogen-Host Interaction (PHI) database, and since the first successful genetic transformation in *B. cinerea* (73), about 150 loss-of-function mutants have been characterized and systematically organized. According to the PHI database, the vast majority (62.73%; 101 genes) of the available *B. cinerea* mutants lead to loss-of-pathogenicity and reduced-virulence phenotypes, with 34.16% of the mutants (55 genes) displaying unaffected pathogenicity and only 5 mutants (3.11%) having an increased virulence (hypervirulence) phenotype (48), a rare but increasingly prevalent infection phenotype among microbial pathogens (74). Any putative mechanistic connection among these five genes (75–78) remains, until today, just speculation, although four out of these five mutants exhibited reduced or impaired conidium production. Interestingly, one of these genes (BcFKBP12) has been implicated in sulfur regulation (75), but its connection with iron metabolism and/or uptake is unknown.

**FIG 8:**
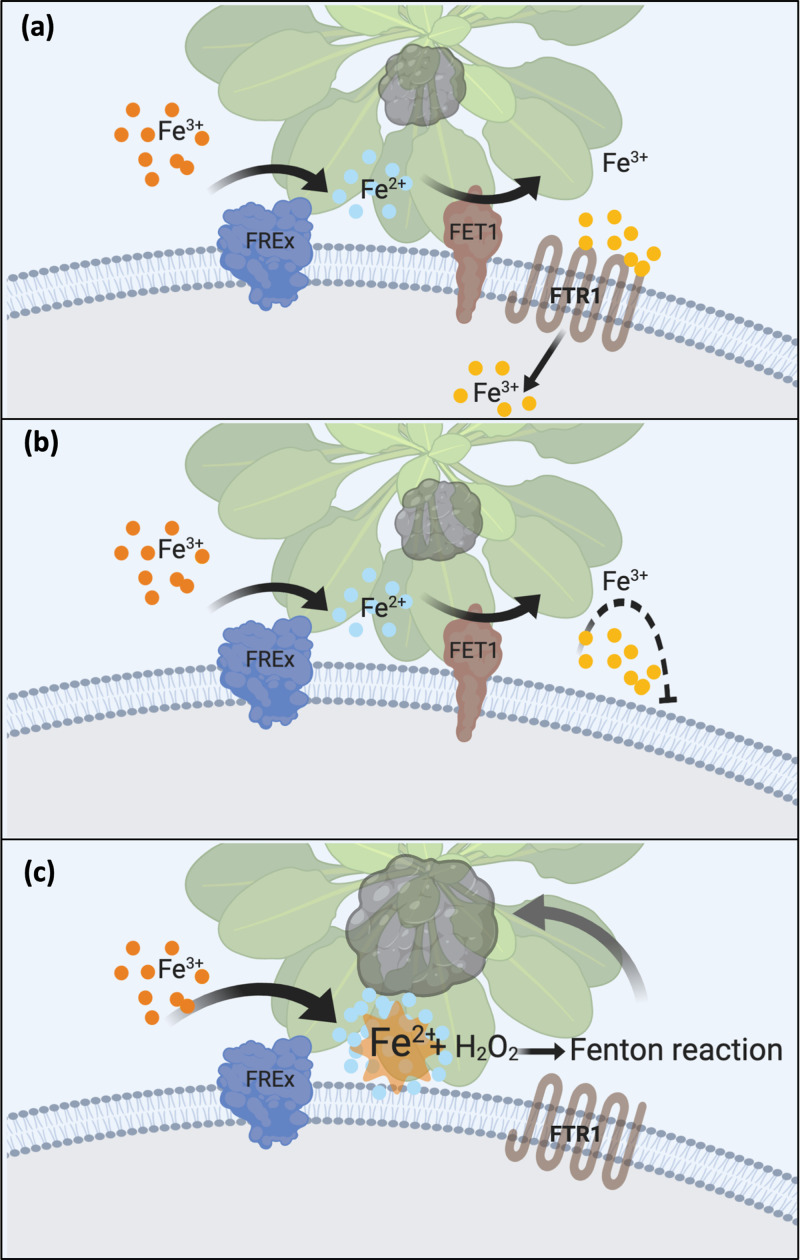
Model of Δ*bcfet1*-dependent hypervirulence in *B. cinerea*. (a) The reductive iron assimilation (RIA) system of *B. cinerea* is mainly composed of BcFET1 (brown membrane-anchor protein) and BcFTR1 (seven transmembrane helices protein). Environmental (chelated) Fe^3+^ (orange dots) is reduced by iron reductases (FREx; blue). The genome of *B. cinerea* encodes at least four of these membrane-bound proteins, producing Fe^2+^ that is subsequently oxidized by BcFET1 (Fe^3+^; yellow dots). Finally, BcFTR1 incorporates iron into the intracellular compartment. A wild-type level of infection is depicted as a gray lesion over the plant’s surface. (b) In the absence of BcFTR1, the ferroxidase BcFET1 produces Fe^3+^ (yellow) that can no longer be incorporated as described for panel a due to the absence of the iron permease. A wild-type level of infection is observed. (c) In the context of the plant defensive oxidative burst and H_2_O_2_ generation, the absence of BcFET1 favors the iron chemical equilibrium toward the reduced form of the metal, generating a stronger (Fenton) oxidative response and the hypervirulence phenotype of infection.

It is logical to expect from an iron acquisition mutant a defect in the incorporation of this metal, as demonstrated here, and, concomitantly, reduced growth. If such a growth defect is observed in a pathogen, it is not easy to distinguish between reduced growth from decreased virulence, since the latter could be just the consequence of the former. Although to extrapolate *in vitro* growth (not altered in this study, at least under the conditions tested) to *in planta* growth behavior should be considered cautiously (e.g., virulence factors are expressed during the infection), it is fascinating to imagine a hypervirulence scenario caused by a growth-impaired mutant. In this regard, a reduction in the cotton blue staining was observed in iron-deprived plants for all three *B. cinerea* strains. In comparison, the two RIA mutant strains developed similar cotton blue staining when infecting replete iron plants (as seen in Fig. 7), reinforcing the interpretation of the enhanced virulence phenotype displayed solely by the lack of one of the RIA components. Thus, the key question is why does only the Δ*bcfet1* strain, and not the Δ*bcftr1* strain, display a hypervirulence phenotype, producing larger necrotic lesions? Although RIA relies on and has been traditionally seen as an interdependent two-component system (the ferroxidase and the iron permease) (28), ferric reductases are needed to remove iron from siderophores (79), not only to provide Fe^2+^, especially under aerobic conditions, to the FET3 ferroxidase that produces Fe^3+^, which is subsequently incorporated by the FTR1 iron permease (Fig. 8a). In the absence of *bcfet1*, one or more iron reductases (the genome of *B. cinerea* encodes at least four of these proteins that are membrane bound) could favor the iron chemical equilibrium toward the reduced form of the metal, which may generate a stronger oxidative response due to the Fenton reaction (24). In the context of the plant oxidative burst associated with the defense response, this scenario may even be facilitated by the production of H_2_O_2_ in a fungal infection that can completely or partially overcome this oxidative response (49, 59) to finally take advantage of ROS, facilitating the production of necrotic lesions (Fig. 8c). In contrast, in the absence of *bcftr1* ROS is not favored, since the ferroxidase still produces the oxidized form of the metal that can no longer be incorporated due to the absence of the permease (Fig. 8b).

As demonstrated here, a loss-of-function mutant unexpectedly displays a significantly increased virulence phenotype, providing us with a unique opportunity to identify new virulence determinants in this genetic background. Genome-wide transcriptomics experiments are being carried out that deal with both defense and infection strategies, as well as the analysis of other iron acquisition components, such as membrane-bound iron reductases, to determine if this is the case.

## MATERIALS AND METHODS

### *B. cinerea* strain and culture conditions.

*B. cinerea* Pers. Fr. (*Botryotinia fuckeliana* [de Bary] Whetzel) strain B05.10 was isolated initially in Germany from a Vitis vinifera vineyard (80). This strain was used as the recipient for genetic modification. Its genome sequence was initially published (4) and significantly improved as a final gapless high-quality genome assembly (81).

The *B. cinerea* B05.10 wild-type strain, as well as genetically modified strains, were cultivated in petri plates (100 mm in diameter) containing one of the following agar-containing media: potato dextrose agar (PDA; Becton, Dickinson) with and without 10% homogenized bean leaves (termed PDAB; cultivated in greenhouses) or Gamborg B5 (Duchefa Biochemie) supplemented with 2% glucose, employed during the genetic transformation procedure (see below). Depending on specific culture conditions, PDA plates were also supplemented with sterile FeCl_3_ (in a range between 100 and 750 μM). Since plants (see below) were routinely cultivated by employing a defined photoperiod, *B. cinerea* strains were also incubated in a 12:12-h light:dark regimen at 20°C using Percival incubators, as described previously (13).

### Cloning of gene replacement and complementation cassettes.

Two replacement cassettes (for Δ*bcfet1* and *Δbcftr1*) were assembled using yeast recombinational cloning (YRC), as described previously (82). Accordingly, the 5′- and 3′-noncoding regions of *bcfet1* and *bcftr1* were PCR amplified from the genomic DNA of *B. cinerea* B05.10 using the primer pairs indicated in Fig. S3 and S8. The hygromycin (*hph*) resistance cassette was amplified from the pCSN44 plasmid (obtained from the Fungal Genetics Stock Center [83]). Primers employed for these PCRs (indicated in the supplemental material) contained 30-bp 5′-overhang regions to allow homologous recombination by YRC. Fragments were cotransformed with the linearized pRS426 vector (84) into uracil-auxotrophic S. cerevisiae strain BY4741 for assembly (85). After YRC, each plasmid containing the desired genetic construct was recovered from individual yeast colonies by Escherichia coli (DH5α) transformation. In the case of the *bcfet1* gene complementation construct (see below), the open reading frame (ORF) of the mentioned gene was amplified from genomic DNA using the primer pair indicated in Fig. S4 (oligonucleotides pc05 and pc59), targeting the “wild-type” Δ*bcfet1* locus and employing the nourseothricin (*nat*) resistance cassette (obtained from the pNR1 vector [86]). The entire genetic construct was sequenced to confirm the absence of mutations. Thereafter, in each case, the replacement/complementation cassettes were PCR amplified using the external primers flanking each construct and the Kapa HiFi DNA polymerase (Kapa Biosystems). These PCR products were used for *B. cinerea* transformation.

### Generation of *B. cinerea* deletion and complemented mutants.

Protoplast generation and transformation of *B. cinerea* were performed as described previously (87) based on the original transformation protocol (73). In brief, *B. cinerea* conidia from a 1-week-old PDAB culture were incubated for 18 h at 20°C and 120 rpm in 100 ml malt extract medium (1.5%). After this period of cultivation, protoplasts from the young mycelia were generated using an enzymatic mixture containing 100 mg of lysing enzyme (Sigma) and 0.5 mg of yatalase (TaKaRa). The obtained protoplasts were mixed with 30 μl of purified PCR products (mentioned above) in polyethylene glycol (PEG) solution (25% PEG 3350, 1 M CaCl_2_, 1 M Tris-HCl, pH 7.5). After 20 to 24 h of fungal regeneration on SH agar, they were overlaid with SH agar containing 70 μg/ml hygromycin B (Invitrogen). Homokaryotic derivates were achieved after iterative steps of single-spore isolation on Gamborg B5–2% glucose supplemented with 70 μg/ml hygromycin B and subsequent transfer of single colonies to new petri dishes. Following DNA extraction (88), transformants that have undergone homologous integration were confirmed by PCR using locus-specific primers (indicated in each corresponding figure in the supplemental material; mentioned above) in combination with *hph*-specific primers. The absence of wild-type alleles was confirmed by using “inner” primers designed to amplify the substituted genomic region, as depicted in Fig. S3, S4, and S8. For *bcfet1* complementation of the Δ*bcfet1* mutant strain, protoplasts of the deletion mutant were transformed with the genetic construct depicted in Fig. S4 and overlaid with SH agar containing 140 μg/ml nourseothricin (Goldbio). Targeted integration of the construct at the *bcfet1* locus was detected using locus-specific primers, as well as nourseothricin (*nat*) resistance cassette-specific oligonucleotides.

### Virulence assays employing Arabidopsis thaliana and Phaseolus vulgaris.

Virulence assays were conducted as described previously (13). Essentially, conidia were suspended in Gamborg B5-2% glucose medium (B5) and adjusted to a final concentration of 2 × 10^5^ conidia/ml in B5 with 10 mM KH_2_PO_4_-K_2_HPO_4_, pH 6.4. Conidial suspensions (7.0 μl) were used to inoculate leaves of Arabidopsis thaliana (Col-0) or primary leaves of French bean (Phaseolus vulgaris cv. Venus from the Instituto Nacional de Investigaciones Agropecuarias [INIA]-Chile). Approximately 5-week-old *Arabidopsis* plants were used. *Arabidopsis* plants were propagated in a 12:12-h light:dark regimen at 20°C using Percival incubators as described previously (13), while P. vulgaris plants were grown for 8 days with artificial LED light employing the mentioned photoperiod at 22°C inside a temperature-controlled growth chamber. For virulence assays, plants were incubated inside plastic boxes at 20°C under a humid environment within Percival incubators for 3 days. When indicated, the commercial substrate green power complete mix (TopCrop) was used, arranged in round plastic planters. This substrate is composed of 35% humus, 10% perlite, 10% vermiculite, 30% coconut fiber, and 15% blond peat. The developed lesions were recorded with a digital camera 72 h postinoculation (hpi) on both *Arabidopsis* and bean leaves. The size of each lesion was calculated by employing a semiautomated procedure using the ImageJ software (89) and an external calibration scale.

To assess the influence of iron on the infection caused by *B. cinerea*, *A. thaliana* plants were grown hydroponically to properly control iron availability. For this purpose, *Arabidopsis* plants were grown in a hydroponic medium for 5 weeks (50 μM FeNa-EDTA) to allow normal development and then washed with 0.30 mM BPDS (bathophenanthroline disulfonic acid). The details of the mentioned strategy were previously described and were performed accordingly (47). In short, 5-day-old seedlings of *A. thaliana* were transplanted from petri dishes containing solid MS medium to an inert substrate (wool rock; GRO-SLAB) moistened with the following hydroponic medium: 0.25 mM Ca(NO_3_)_2_ × 4H_2_O, 1 mM KH_2_PO_4_, 0.5 mM KNO_3_, 1 mM MgSO_4_ × 7H_2_O, 50 μM H_3_BO_3_, 19 μM MnCl_2_ × 4H_2_O, 10 μM ZnCl_2_, 1 μM CuSO_4_ × 5H_2_O, 0.02 μM Na_2_MoO_4_ × 2H_2_O, and 50 μM FeNa-EDTA. After 5 weeks, roots were washed with distilled water and BPDS as described previously (47) and transferred back to a fresh hydroponic medium without iron. On the other hand, the control group was subjected to the same washing treatment but without BPDS, transferring these plants to fresh hydroponic medium containing 50 μM FeNa-EDTA. Plants were incubated for 4 days under the conditions just described before performing virulence assays.

### Real-time qPCR.

PCR-verified mutants were further analyzed by qPCR to determine the number of *hph* cassette insertions. For this purpose, two pairs of primers were used (Table 1) that amplify a defined and specific segment of the *bcfrq1* gene of *B. cinerea* and *hph*. The *B. cinerea bcfrq1* gene was chosen because it is a single-copy gene in the genome whose qPCR primers display very high qPCR amplification efficiency, as previously demonstrated (13) and validated here (Table 1). For both pairs of primers, each corresponding PCR amplicon was synthesized *in vitro* as a single-strand DNA molecule (Table 1; denoted amp97-98 and amp32-687). Employing known concentrations of these single-strand DNA templates (or amplicons) and 1:10 serial dilutions, the qPCR calibration curves were constructed as recommended (90), starting in each case from 500,000 molecules/μl. Parallel qPCRs, employing genomic DNA derived from each analyzed mutant strain, were also performed. The qPCR crossing point (Cp) values obtained for the genomic DNA samples of each mutant isolate were interpolated into the mentioned calibration curves to determine the number of amplified DNA molecules, thereby defining the *hph*/*bcfrq1* ratio (Table 2). For the qPCRs, the commercial SYBR FAST qPCR system (Kapa Biosystems) was used according to the manufacturer’s directions. The qPCRs were carried out in the AriaMx real-time PCR system (Stratagene) using the following amplification profile: initial denaturation at 95°C during 60 s, followed by 40 two-step qPCR of 5 s at 95°C and 15 s at 62°C.

**TABLE 1:** Oligonucleotides employed in qPCR analysis

Target or qPCR amplicon	Primer^*a*^ (5′–3′)	Primer name	Amplicon size (bp)	Cq		Efficiency		
				Lower level	Higher level	%	Curve (*R*^2^)	Curve order of magnitude
*bcfrq1*	FW, ACCCAGGAGGAAAGGTACGAA	oL97	86	13.44 ± 0.01	26.39 ± 0.76	98.33	0.995	6
	RC, GGGAGCGGAAGGACAGATTT	oL98						
*hph*	RC, GATTTCAGTAACGTTAAGTGG	oL687	111	14.12 ± 0.01	30.64 ± 0.34	98.95	0.997	6
	FW, ATGGCTGTGTAGAAGTACTC	oL32						
*bcfrq1* amplicon	ACCCAGGAGGAAAGGTACGAAATGGAAGCCAGCGACAAGAGTGTTCAGTTGGGGAGTG CGGCGAGTAAATCTGTCCTTCCGCTCCC	amp97-98						
*hph* amplicon	GATTTCAGTAACGTTAAGTGGATCCCGGTCGGCATCTACTCTATTCCTTTGCCCTCGGACGAGTGCTGGGGCGTCGGTTTCCACTATCGGCGAGTACTTCTACACAGCCAT	amp687-32						

a FW, forward orientation; RC, reverse orientation.

**TABLE 2:** Determination of copy insertions of the *hph* cassette in each analyzed mutant strain^*a*^

*B. cinerea* mutant isolate	*hph*/*scg* ratio (molecules)
Δ*bcfet1-1*	1.11 ± 0.09
Δ*bcfet1-2*	1.30 ± 0.08
Δ*bcfet1-3*	1.09 ± 0.04
Δ*bcftr1-1*	1.10 ± 0.06
Δ*bcftr1-2*	1.04 ± 0.04

a Shown is the copy number of *hph* referring to the single-copy gene (*scg*) *bcfrq1*.

### Determination of iron content in whole-cell fungal tissue.

To determine the iron content in whole-cell fungal tissue, B05.10, Δ*bcfet1*, and Δ*bcftr1* strains were grown on petri dishes containing PDA medium covered with cellophane paper to allow easy recovery of the fungal material. Tissue was collected in 2.0-ml Eppendorf tubes after 3 dpi. The fungal tissue was lyophilized until fully dried at −44°C and 9 Pa and subjected to weight determination. Completely dehydrated samples were digested and homogenized in 1.0 ml of 3% nitric acid during 16 h at 95°C by following the previously described procedure (91). The iron content present in the solution was subsequently determined by an atomic absorption spectrometer (Shimadzu AA-7000) employing an iron(III) (FeCl_3_) calibration curve (Sigma).

### DAB and cotton blue staining.

H_2_O_2_ levels *in planta* were determined by applying 3,3′-diaminobenzidine (DAB) staining as described previously (51). Briefly, detached leaves were incubated in a 2 mM EDTA solution, pH 5.5, and subsequently incubated in a 5 mM DAB solution, pH 3.8, during 2 h with agitation. Leaves were destained in lactophenol. An additional cotton blue staining then was performed, allowing the detection of chitin, a polysaccharide that is present in the fungal cell wall (52, 92). After that, images were acquired using an Olympus CX21 microscope attached to a digital camera.

### Computational and statistical analyses.

Phylogenetic reconstruction of MCO proteins encoded by the *B. cinerea* genome was performed by employing the one-click method of the Phylogeny.fr platform, applying default settings (42). For comparative purposes, several filamentous fungal RIA-related MCO ferroxidases were obtained from the fungal genomes of Aspergillus flavus, Claviceps purpurea, Colletotrichum orbiculare, Fusarium graminearum, Magnaporthe oryzae, Penicillium digitatum, Sclerotinia sclerotiorum, Trichoderma reesei, *U. maydis*, Verticillium dahliae, and Zymoseptoria tritici, available from FASTA files at Ensembl Fungi (93). Manual analysis and inspection of sequences were performed using ClustalW (94). GraphPad Prism software (version 8.3.0) was used for statistical analysis and data representation. Analysis of variance and Tukey’s post test were performed (*P* ≤ 0.05). A working model (depicted in Fig. 8) was constructed with the BioRender.com software.
