# Supplementary figures and images for: Defects in the Ferroxidase That Participates in the Reductive Iron Assimilation System Results in Hypervirulence in Botrytis Cinerea
Source: mBio. 2020 Aug 4;11(4):e01379-20. doi: 10.1128/mBio.01379-20 (PMC7407086; doi:10.1128/mBio.01379-20)

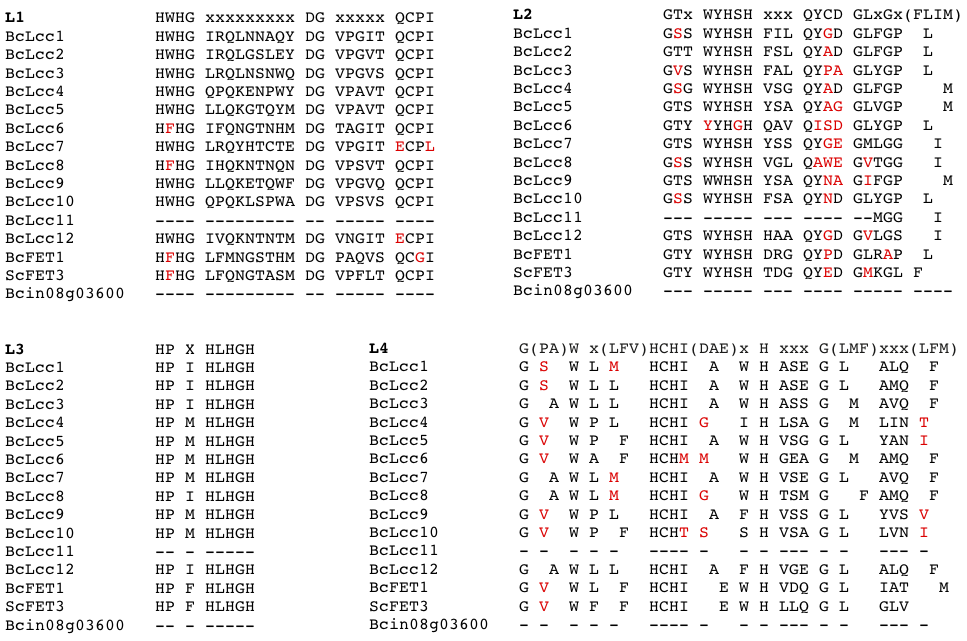

Supplement: FIG S1 [file mBio.01379-20-sf001.tif]

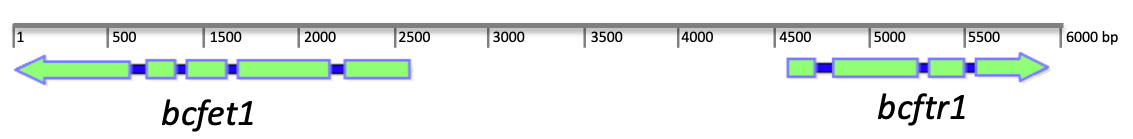

Supplement: FIG S2 [file mBio.01379-20-sf002.tif]

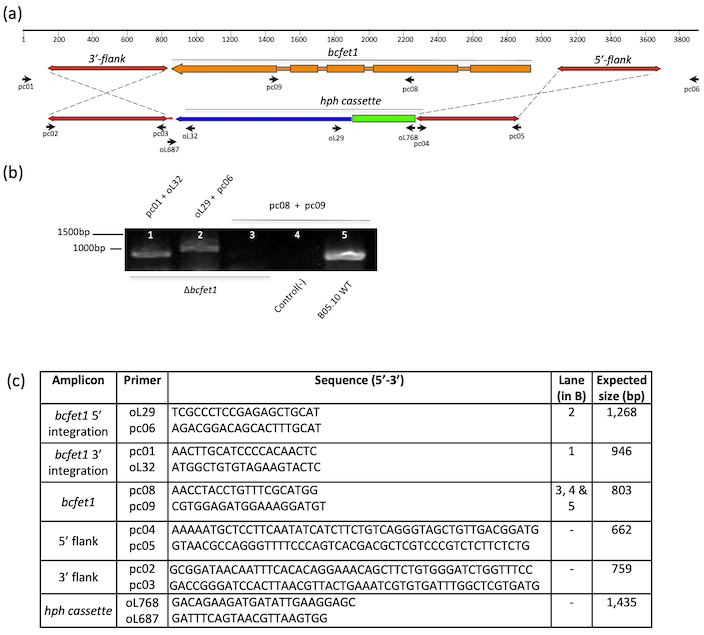

Supplement: FIG S3 [file mBio.01379-20-sf003.tif]

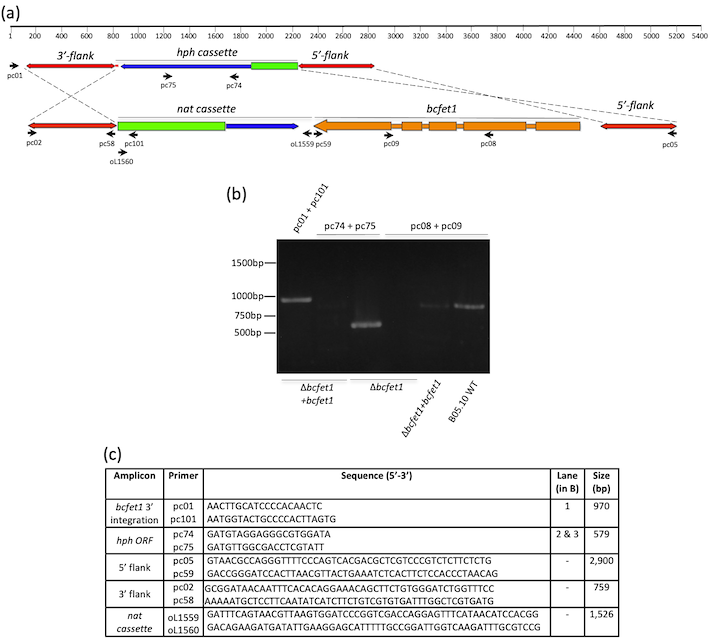

Supplement: FIG S4 [file mBio.01379-20-sf004.tif]

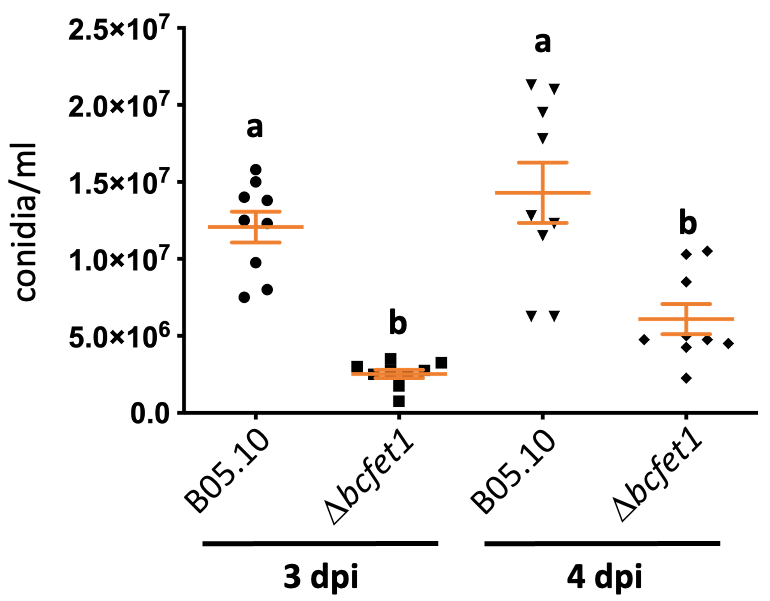

Supplement: FIG S5 [file mBio.01379-20-sf005.tif]

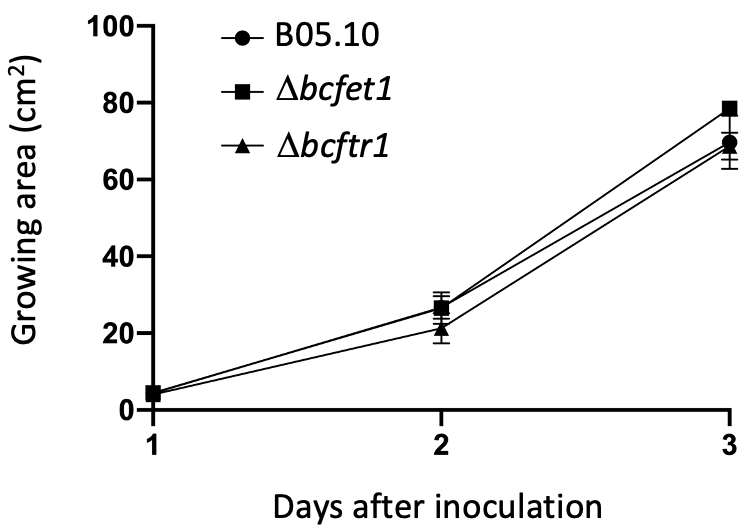

Supplement: FIG S6 [file mBio.01379-20-sf006.tif]

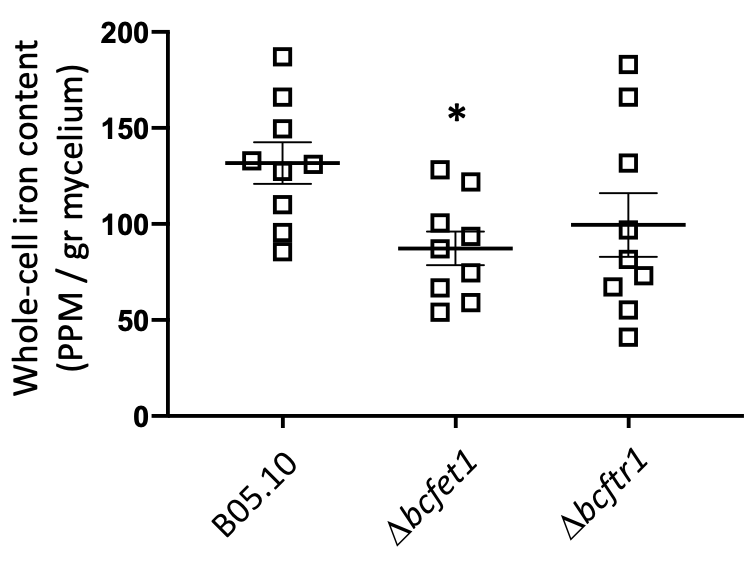

Supplement: FIG S7 [file mBio.01379-20-sf007.tif]

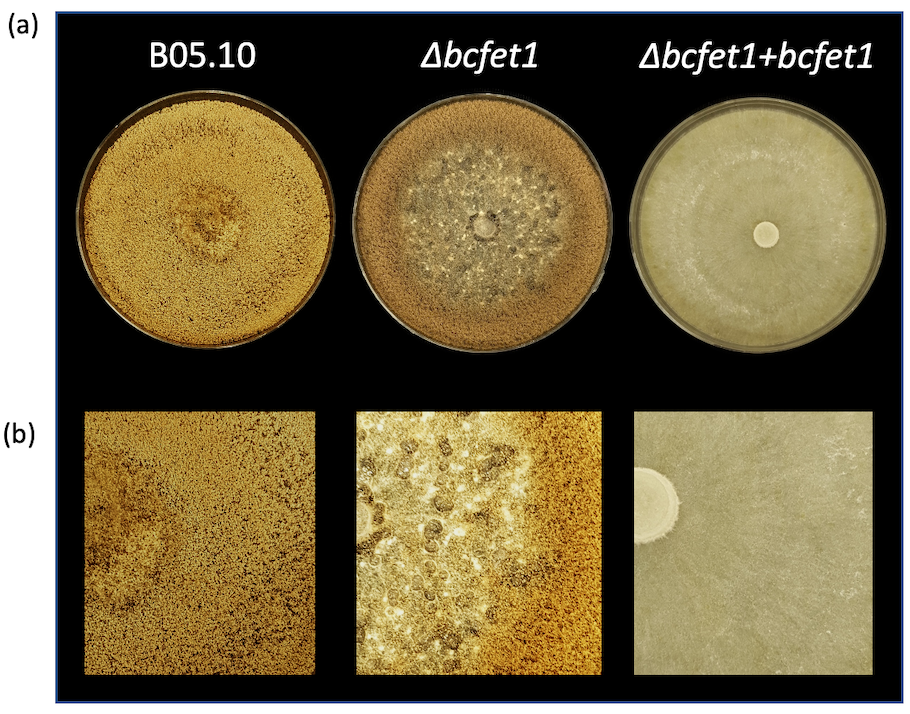

Supplement: FIG S8 [file mBio.01379-20-sf008.tif]

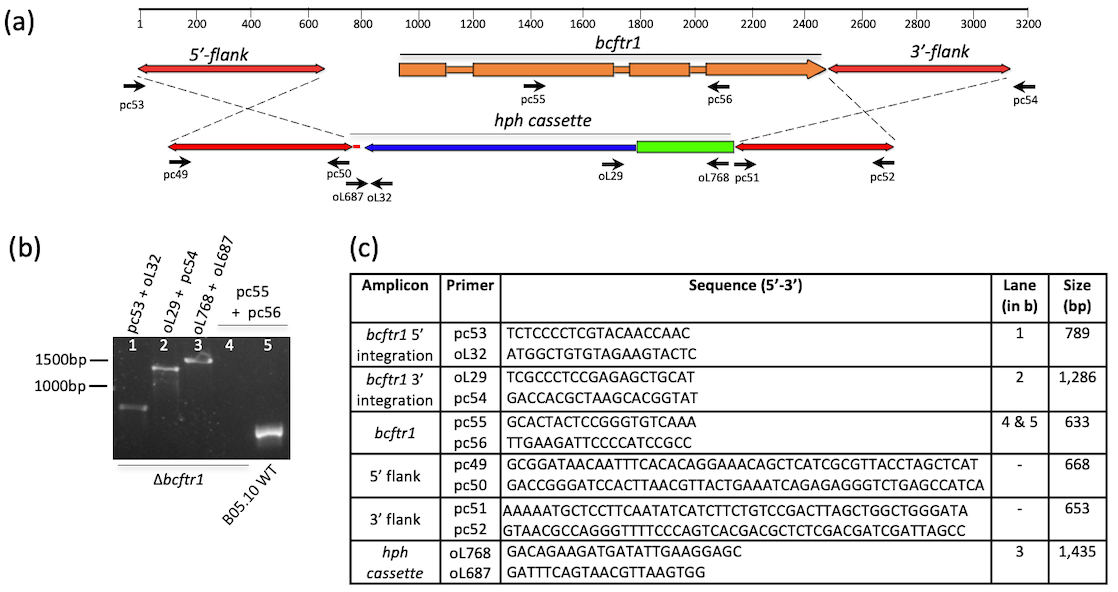

Supplement: FIG S9 [file mBio.01379-20-sf009.tif]
