# Supplementary material for: Defects in the Ferroxidase That Participates in the Reductive Iron Assimilation System Results in Hypervirulence in Botrytis Cinerea
Source: mBio. 2020 Aug 4;11(4):e01379-20. doi: 10.1128/mBio.01379-20 (PMC7407086; doi:10.1128/mBio.01379-20)
Supplement: TABLE S1 [file mBio.01379-20-st001.docx]

| **Gene ID** | **name** | **paralogs / observations** | **# of aa** | **PFAM** |
| --- | --- | --- | --- | --- |
| Bcin01g07950 | *bclcc1* | 9 paralogs | 565 | MCO, PFAM PF00394, PF07731, PF07732 |
| Bcin14g02510 | *bclcc2* | 9 paralogs | 581 | MCO, PFAM PF00394, PF07731, PF07732 |
| Bcin09g04830 | *bclcc3* | 9 paralogs | 602 | MCO, PFAM PF00394, PF07731, PF07732 |
| Bcin05g03550 | *bclcc4* | 9 paralogs | 587 | MCO, PFAM PF00394, PF07731, PF07732 |
| Bcin01g07190 | *bclcc5* | 9 paralogs | 593 | MCO, PFAM PF00394, PF07731, PF07732 |
| Bcin15g03330 | *bclcc6* | NO paralogs predicted | 734 | MCO, PFAM PF00394, PF07731, PF07732 |
| Bcin02g07640 | *bclcc7* | 9 paralogs | 663 | MCO, PFAM PF00394, PF07731, PF07732 |
| Bcin01g00800 | *bclcc8* | 9 paralogs | 710 | MCO, PFAM PF00394, PF07731, PF07732 |
| Bcin07g06780 | *bclcc9* | 9 paralogs | 589 | MCO, PFAM PF00394, PF07731, PF07732 |
| Bcin09g02050 | *bclcc10* | 9 paralogs | 604 | MCO, PFAM PF00394, PF07731, PF07732 |
| Bcin08g00050 | *bclcc11* | NO paralogs predicted | 124 | only type 1 MCO, PFAM PF00394 |
| Bcin13g00110 | *bclcc12* | 9 paralogs | 607 | MCO, PFAM PF00394, PF07731, PF07732 |
| Bcin02g02780 | *bclcc13;* termed herein *bcfet1* | termed *bcfet1*, Bcin02g02790: iron permease (*bcftr1*); NO paralogs predicted | 615 | MCO, PFAM PF00394, PF07731, PF07732 |
| Bcin08g03600 | NO name | NO paralogs predicted | 96 | only type 1 MCO, PFAM PF00394 |

**Table S1.**
